# Sub-clinical thickening of the fovea in diabetes and its relationship to glycaemic control: a study using swept-source optical coherence tomography

**DOI:** 10.1007/s00417-020-04914-2

**Published:** 2020-09-08

**Authors:** Ross T. Aitchison, Graeme J. Kennedy, Xinhua Shu, David C. Mansfield, Uma Shahani

**Affiliations:** 1 School of Health and Life Sciences, Glasgow Caledonian University, Glasgow, UK; 2 Department of Ophthalmology, Inverclyde Royal Hospital, Greenock, UK

**Keywords:** Foveal thickness, Sub-clinical thickening, Diabetic cystoid macular oedema, Glycated haemoglobin, Swept-source optical coherence tomography

## Abstract

**Background:**

Accumulation of multiple pockets of fluid at the fovea, as a complication of poor blood glucose control in diabetes, causes impairment of central vision. A new ability to demonstrate a pre-clinical phase of this maculopathy could be valuable, enabling diabetic individuals to be alerted to the need to improve their glycaemic control. This study aimed to use swept-source optical coherence tomography (SS-OCT) to measure foveal thickness and macular volume in diabetic individuals without cystoid macular oedema, and in non-diabetic individuals, and relate these measures to participants’ glycaemic control.

**Methods:**

Centre point thickness (CPT) and total macular volume (TMV) were measured using SS-OCT (DRI OCT Triton™, Topcon, Tokyo, Japan). Participants’ glycosylated haemoglobin (HbA_1c_) level was also assessed (A_1c_Now®+ System, PTS Diagnostics, Indianapolis, IN, USA). The diabetic (*n* = 27) and non-diabetic (*n* = 27) groups were matched for age (*p* = 0.100) and sex (*p* = 0.414), and HbA_1c_ level differed between diabetic and non-diabetic groups (*p* < 0.0005). The diabetic group comprised type 1 (*n* = 7) and type 2 (*n* = 20) diabetic individuals who were matched for duration of diabetes (*p* = 0.617) and whose glycaemic control was similar (*p* = 0.814).

**Results:**

Diabetic individuals had significantly higher CPT (*t*(37) = 3.859, *p* < 0.0005) than non-diabetic individuals. In the diabetic group, multiple linear regression analysis revealed a conspicuous relationship between CPT and HbA_1c_ level (*β* = 0.501, *t*(21) = 3.139, *p* = 0.005): there was a 19-μm increase in CPT for each 1% increase in HbA_1c_ level. This relationship was not present in the non-diabetic group (*β* = − 0.068, *t*(23) = − 0.373, *p* = 0.712).

**Conclusions:**

SS-OCT is the only way to measure macular thickness *in vivo*. Diabetic individuals en bloc had higher CPT compared with non-diabetic individuals. Moreover, in the diabetic group, HbA_1c_ level significantly predicted CPT. Our results suggest that, in diabetes, sub-clinical thickening may occur at the fovea before cystoid macular oedema becomes clinically evident. This could provide diabetic individuals with an early warning of disease progression and motivate them to improve control of their diabetes, with a view to avoiding the need of intra-vitreal injections with their attendant risks.
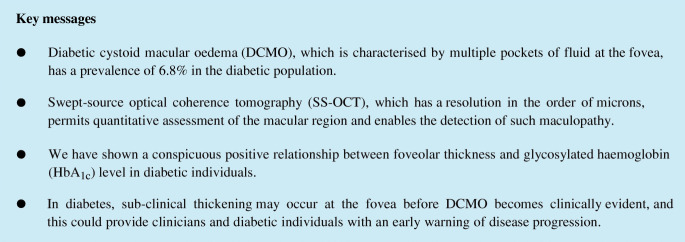

## Introduction

Diabetic cystoid macular oedema (DCMO), which is characterised by multiple pockets of fluid at the fovea, is the leading cause of visual impairment in people of working age [1]. It is a complication of poor blood glucose control in diabetes and has a prevalence of 6.8% in the diabetic population [2].

A number of large-scale studies have found that tight glycaemic control is effective in decreasing the incidence of diabetic complications [3–6]. Glycosylated haemoglobin (HbA_1c_) is the gold-standard method used to assess long-term glycaemic control. Glucose is added to the N-terminal end of haemoglobin molecules irreversibly by enzymically catalysed glycosylation at a rate that is proportional to glucose concentration in the blood [7]. Erythrocytes have a lifespan of 3 months, and HbA_1c_ is broken down when these cells are destroyed in the liver and spleen; therefore, the proportion of haemoglobin that is glycosylated serves as a measure of plasma glucose in the preceding 3-month period. The reference range for HbA_1c_ in an adult without diabetes is 4.0–5.9% [8], and a value greater than 6.5% is the diagnostic criterion for diabetes [9].

The retina is particularly susceptible to microvascular damage in diabetes because of its high metabolic and oxygen demands, and its dependence on the integrity of the blood–retinal barrier (BRB) [10]. Although the pathophysiology of DCMO is multifactorial and complex, loss of pericytes [11–13] and alteration in the amount of vascular endothelial growth factor [14] are known to play a key role in the onset of the condition. The exact mechanisms by which hyperglycaemia leads to diabetic retinopathy (DR) and DCMO remain poorly defined; however, several biochemical pathways have been implicated and tested in the pathogenesis of DR [15] and DCMO [14]. The BRB comprises two components: the inner BRB and outer BRB. The inner BRB is formed by tight junctions between the endothelial cells of the retinal capillaries; the outer BRB is formed by tight junctions between the retinal pigment epithelial cells. When the integrity of the BRB is impaired, proteins and lipids leak into the interstitial space. This causes an increase in oncotic pressure, whereby fluid is drawn out of the vessels. Depending on whether it is the inner or outer component of the BRB that is damaged, accumulation of intra- and sub-retinal fluid respectively may ensue [16]. Breakdown of the BRB in the clinical environment has been assessed using fundus fluorescein angiography and vitreous fluorometry [17], both of which require invasive intra-venous administration of fluorescein.

In recent years, optical coherence tomography (OCT), a non-invasive imaging technique that uses low-coherence light interference to produce high-resolution images of the retinal structure in vivo, has been used to assess DCMO. OCT permits cross-sectional visualisation of the macular region. Since its inception in 1991 [18], there have been significant advancements in OCT, insofar as it is now possible for the macular region to be assessed objectively and, therefore, for the progression of DCMO to be monitored quantitatively [19]. The latest generation of this technology, swept-source optical coherence tomography (SS-OCT), uses a higher wavelength of light than that used in previous generations, permitting an increased depth of imaging. Moreover, the increased scanning speed of SS-OCT reduces the likelihood of motion artefacts, and its axial resolution is in the order of microns.

The aim of the study described in this report was to use SS-OCT to evaluate foveal thickness and macular volume in diabetic individuals with no clinically significant macular oedema, and in non-diabetic individuals, and relate these measures to participants’ glycaemic control.

## Methods

### Participants

Fifty-four participants took part in this study, all of whom were Caucasian. Participants were recruited from the Vision Centre at Glasgow Caledonian University, and informed written consent was obtained from all participants prior to their participation in the study.

In the diabetic group (*n* = 27), the mean age (± SD (standard deviation)) was 55 ± 19 years, and the male-to-female ratio was 15:12. In the non-diabetic group (*n* = 27), the mean age was 42 ± 23 years, and the male-to-female ratio was 11:16. Diabetic and non-diabetic groups were matched for age (*U* = 270, *z* = − 1.645, *p* = 0.100) and sex (*χ*^2^ (1, *n* = 54) = 0.276, *p* = 0.414).

The mean HbA_1c_ level (± SD) was 7.5 ± 0.8% in the diabetic group, and the range was from 6.1 to 9.0%. In the non-diabetic group, the mean HbA_1c_ level was 5.4 ± 0.4%, and the range was from 4.7 to 5.9%. HbA_1c_ was normally distributed in both the diabetic group (*W*(27) = 0.970, *p* = 0.593) and non-diabetic group (*W*(27) = 0.925, *p* = 0.052). This between-group difference was statistically significant (*t*(39) = 8.853, *p* < 0.0005); as the variances were unequal (*F* = 12.805, *p* = 0.001), the degrees of freedom were adjusted from 52 to 39.

The diabetic group comprised individuals with type 1 diabetes (*n* = 7) and type 2 diabetes (*n* = 20). Compared with participants in the type 1 diabetic sub-group (mean age (± SD) *=* 30 ± 18 years), participants in the type 2 sub-group (mean age *=* 64 ± 9 years) were significantly older (*U* = 17, *z* = − 2.934, *p* = 0.003). However, the duration of diabetes in the type 1 diabetic sub-group (mean (± SD) *=* 11 ± 9 years) and type 2 diabetic sub-group (mean *=* 12 ± 7 years) was similar (*U* = 61, *z* = − 0.501, *p* = 0.617). HbA_1c_ level in the type 1 diabetic sub-group (mean (± SD) *=* 7.4 ± 0.6%; *W*(7) = 0.932, *p* = 0.567) and type 2 diabetic sub-group (mean *=* 7.5 ± 0.8%; *W*(20) = 0.963, *p* = 0.597) was also similar (*t*(25) = 0.237, *p* = 0.814).

### Inclusion and exclusion criteria

All participants had a best-corrected visual acuity of 0.3 logMAR or better in each eye, and the inter-ocular difference in visual acuity was no greater than one line (0.1 logMAR). Participants in the diabetic group had been diagnosed with either type 1 or 2 diabetes mellitus by a diabetologist. Furthermore, all diabetic participants reported no previous diagnosis of DR or DCMO, and participants whom we found to have DCMO were also excluded. Participants with any ocular disease, such as cataract, age-related macular degeneration or glaucoma, were excluded from the study.

### Glycosylated haemoglobin

Participants’ HbA_1c_ level was measured using the A_1c_Now®+ System (PTS Diagnostics, Indianapolis, IN, USA). A 5-μl capillary blood sample was obtained by means of a single-use lancet. This system used the principle of colourimetry, and test results were expressed as the percentage of total haemoglobin that was glycosylated in the sample. The method by which the A_1c_Now®+ System assesses HbA_1c_ level has been described in detail elsewhere [20].

### Apparatus

SS-OCT (DRI OCT Triton™, Topcon, Tokyo, Japan) was used to take 7 × 7 mm *3D Macula Map* scans. This instrument used a wavelength-sweeping laser with a central wavelength of 1050 nm and a tuning range of approximately 100 nm. The scanning speed of the instrument was 100,000 A-scans/s, and its axial resolution was 2.6 μm.

**Fig. 1:**
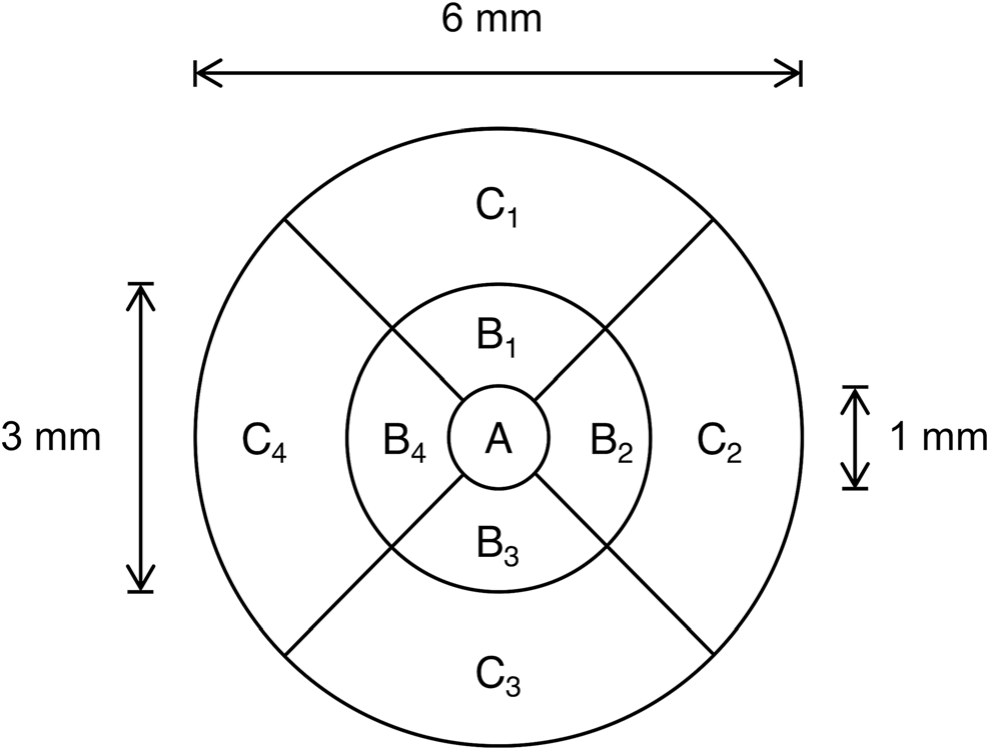
ETDRS grid

Centre point thickness (CPT) was automatically determined by proprietary software and was calculated as the distance between the inner limiting membrane and the outer segment–retinal pigment epithelium interface. CPT was measured at the foveola: the locus of the intersection of the six 7-mm radial scans that comprised the *3D Macula* scan. An Early Treatment of Diabetic Retinopathy Study (ETDRS) grid [21] was centred on this intersection of the radial scans. The ETDRS grid comprised three concentric circles that were 1 mm, 3 mm and 6 mm in diameter, and the grid was divided into nine sub-fields (Fig. 1). Total macular volume (TMV), in mm^3^, was calculated using the mean thickness, in μm, of each sub-field:

$$ \mathrm{TMV}=\frac{\uppi \mathrm{A}}{4000}+\frac{{\uppi \mathrm{B}}_1+{\uppi \mathrm{B}}_2+{\uppi \mathrm{B}}_3+{\uppi \mathrm{B}}_4}{2000}+\frac{27\left({\uppi \mathrm{C}}_1+{\uppi \mathrm{C}}_2+{\uppi \mathrm{C}}_3+{\uppi \mathrm{C}}_4\right)}{16000} $$

### Statistical methods used

All statistical analyses were performed using the SPSS Statistics 26 (IBM Corp., Armonk, NY, USA). Intra-class correlation analysis was used to assess for inter-ocular differences in CPT and TMV. Independent-sample *t* tests were run to assess for a difference in these macular measures between diabetic and non-diabetic groups. A simultaneous method of multiple linear regression was run to examine the effects of HbA_1c_ level, age and sex (and duration and type of diabetes in diabetic participants) on CPT. Similar analysis was then run to assess the effects of these predictor variables on TMV. Shapiro−Wilk *W* tests were used to assess the normality of distribution, and outliers were assessed by inspection of boxplots for values greater than 1.5 times the interquartile range (IQR). For all statistical tests, parametric assumptions were met, and the alpha-level (*α*) was set at 0.05.

## Results

### Inter-ocular relationship

Using intra-class correlation analysis, we found that measures of CPT and TMV that were obtained from participants’ right and left eyes were similar (Table 1). A two-way random-effects model with single measures and absolute agreement was employed for all measures (ICC (A, 1)) [22]. In accordance with statistical guidelines for data obtained from two eyes [23, 24], because there was a significant inter-ocular relationship for CPT and TMV above parameters, we used the mean value of the right and left eyes for each participant.

**Table 1:** Intra-class correlation analysis between participants’ right and left eyes

	Non-diabetic group	Diabetic group	Type 1 diabetic sub-group	Type 2 diabetic sub-group
CPT	*ρ =* 0.613 ***	*ρ =* 0.632 ***	*ρ =* 0.632 *	*ρ =* 0.572 **
TMV	*ρ =* 0.900 ***	*ρ =* 0.858 ***	*ρ =* 0.911 ***	*ρ =* 0.851 *

The intra-class correlation co-efficient is denoted by *ρ*

^*^p ≤ 0.05

^**^p ≤ 0.01

^***^p ≤ 0.001

### Centre point thickness

**Fig. 2:**
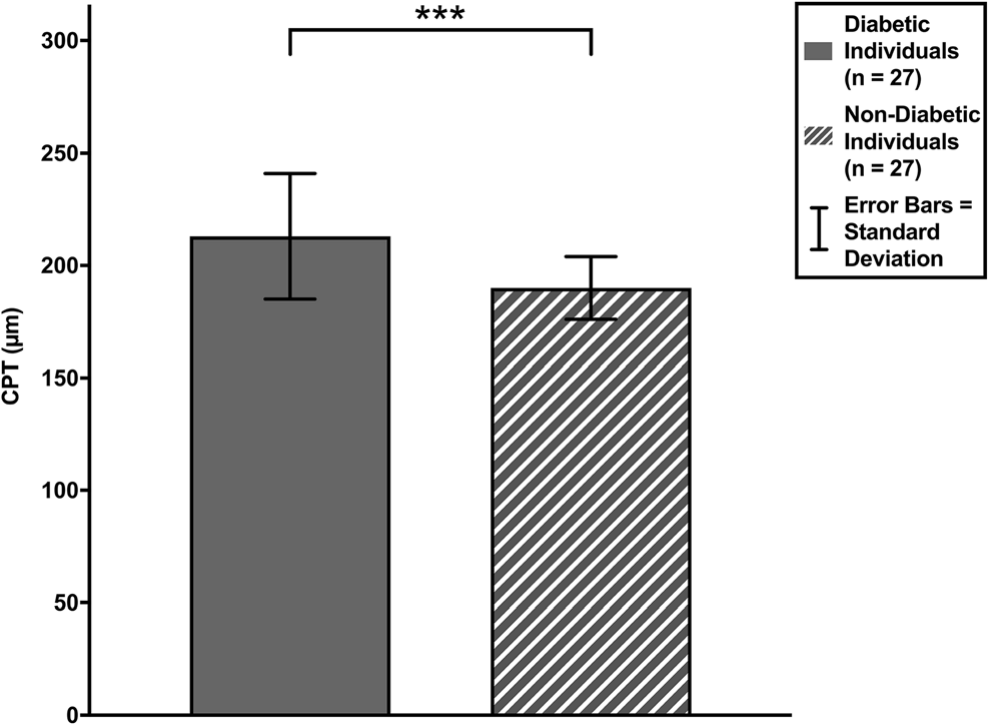
CPT in diabetic and non-diabetic individuals (* *p* ≤ 0.05, ** *p* ≤ 0.01, *** *p* ≤ 0.001)

Firstly, we assessed for a difference in CPT between diabetic and non-diabetic groups. CPT values were normally distributed in both the diabetic group (*W*(27) = 0.961, *p* = 0.383) and non-diabetic group (*W*(27) = 0.979, *p* = 0.841), and there were no outliers in the data. Diabetic individuals had a statistically significantly greater CPT compared with their non-diabetic counterparts (*t*(37) = 3.859, *p* < 0.0005); as the variances were unequal (*F* = 9.102, *p* = 0.004), the degrees of freedom were adjusted from 52 to 37. The mean (± SD) CPT was 213 ± 28 μm in the diabetic group and 190 ± 14 μm in the non-diabetic group (Fig. 2).

**Fig. 3:**
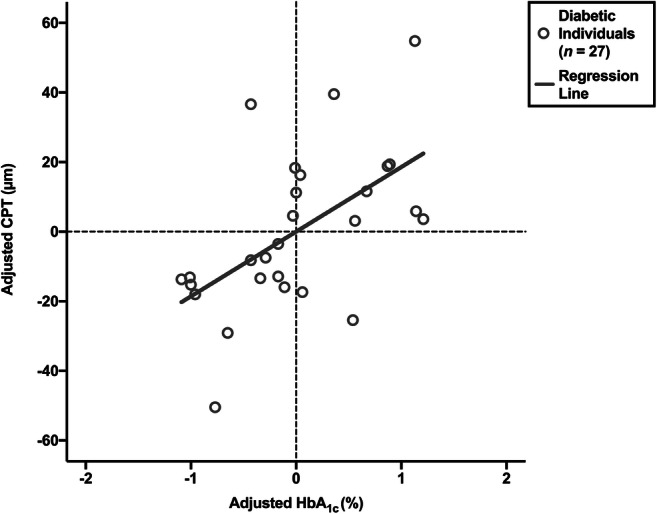
Partial regression plot of CPT against HbA_1c_ in diabetic participants when controlling for age, sex, type of diabetes and duration since diagnosis of diabetes

Multiple linear regression was then used to examine the effects of participants’ HbA_1c_ level, age and sex on their CPT. In addition, the effects of duration and type of diabetes on diabetic participants’ CPT were added to the model. In the diabetic group, the analysis was found to be statistically significant (*F*(5,21) = 5.210, *p* = 0.003, *R*^2^ = 0.554) (Table 2). Similar analysis performed on non-diabetic individuals’ data did not reach statistical significance (*F*(3,23) = 2.813, *p* = 0.062, *R*^2^ = 0.268) (Table 3). In the diabetic group, HbA_1c_ level significantly predicted CPT (*β* = 0.501, *t*(21) = 3.139, *p* = 0.005): there was a 19-μm increase in CPT for every 1% increase in HbA_1c_ level (Fig. 3). there wasno significant relationship between HbA_1c_ level and CPT in the non-diabetic group (*β* = − 0.068, *t*(23) = − 0.373, *p* = 0.712). Age was related to CPT in both the diabetic group (*β* = 0.715, *t*(21) = 2.356, *p* = 0.028) and non-diabetic group (*β* = 0.485, *t*(23) = 2.479, *p* = 0.021). Male participants had increased CPT compared with female participants, and this was the case in the diabetic group (*β* = 0.211, *t*(21) = 1.282, *p* = 0.214) and non-diabetic group (*β* = 0.073, *t*(23) = 0.073, *p* = 0.713); however, these differences did not reach statistical significance. In addition, the duration and type of diabetes were assessed in the diabetic group: participants with type 1 diabetes had greater CPT compared with individuals with type 2 diabetes (*β* = − 0.223, *t*(21) = − 0.834, *p* = 0.414), although this difference did not achieve statistical significance; there was no significant effect of duration of diabetes (*β* = − 0.241, *t*(21) = − 1.239, *p* = 0.229).

**Table 2:** Multiple linear regression analysis of CPT in diabetic participants

CPT	*B*	95% confidence interval		*β*	*t*	*R*^2^	*ΔR*^2^
Diabetic group		Lower limit	Upper limit				
Model						0.554	0.447 **
Constant	42.855	− 50.637	136.347		0.953		
HbA_1c_	18.585 **	6.272	30.898	0.501 **	3.139		
Age	1.049 *	0.123	1.975	0.715 *	2.356		
Sex (M − F)	11.761	− 7.322	30.844	0.211	1.282		
Type (2–1)	− 14.092	− 49.240	21.056	− 0.223	− 0.834		
Duration	− 0.765	− 2.048	0.518	− 0.241	− 1.239		

The un-standardised regression co-efficient is denoted by *B*; the standardised co-efficient is denoted by *β*; the standardised co-efficient divided by its standard error (SE) is denoted by *t*; the co-efficient of determination is denoted by *R*^2^; the adjusted co-efficient of determination is denoted by Δ*R*^2^

^*^p ≤ 0.05

^**^p ≤ 0.01

^***^p ≤ 0.001

**Table 3:** Multiple linear regression analysis of CPT in non-diabetic participants

CPT	*B*	95% confidence interval		*β*	*t*	*R*^2^	*ΔR*^2^
Non-diabetic group		Lower limit	Upper limit				
Model						0.268	0.173
Constant	189.387 ***	118.398	260.376		5.519		
HbA_1c_	− 2.379	− 15.560	10.803	− 0.068	− 0.373		
Age	0.292*	0.048	0.535	0.485*	2.479		
Sex (M − F)	1.973	− 8.996	12.941	0.073	0.372		

The un-standardised regression co-efficient is denoted by *B*; the standardised co-efficient is denoted by *β*; the standardised co-efficient divided by its standard error (SE) is denoted by *t*; the co-efficient of determination is denoted by *R*^2^; the adjusted co-efficient of determination is denoted by Δ*R*^2^

^*^p ≤ 0.05

^**^p ≤ 0.01

^***^p ≤ 0.001

### Total macular volume

TMV values were normally distributed in both the diabetic group (*W*(27) = 0.977, *p* = 0.801) and non-diabetic group (*W*(27) = 0.972, *p* = 0.650), and there were no outliers in the data. The mean (± SD) TMV was 7.62 ± 0.41 mm^3^ in the diabetic group and 7.76 ± 0.37 mm^3^ in the non-diabetic group. TMV did not differ significantly between the diabetic and non-diabetic groups (*t*(52) = − 1.363, *p* = 0.179).

Multiple linear regression was then used to examine the effects of participants’ HbA_1c_ level, age and sex on their TMV; the effects of duration and type of diabetes on diabetic participants’ TMV were also included in the model. The analysis was not statistically significant in the diabetic group (*F*(5,21) = 1.638, *p* = 0.194, *R*^2^ = 0.281) (Table 4) or non-diabetic group (*F*(3,23) = 0.584, *p* = 0.631, *R*^2^ = 0.071) (Table 5). HbA_1c_ did not predict TMV in either group (diabetic group: *β* = 0.072, *t*(21) = 0.357, *p* = 0.725; non-diabetic group: *β* = − 0.012, *t*(23) = − 0.057, *p* = 0.955). Likewise, TMV was not associated with participants’ age (diabetic group: *β* = − 0.569, *t*(21) = − 1.475, *p* = 0.155; non-diabetic group: *β* = − 0.255, *t*(23) = − 1.157, *p* = 0.259) or sex (diabetic group: *β* = − 0.335, *t*(21) = − 1.600, *p* = 0.124; non-diabetic group: *β* = − 0.022, *t*(23) = − 0.102, *p* = 0.920). In the diabetic group, TMV was not affected by the type (*β* = 0.394, *t*(21) = 1.161, *p* = 0.259) or duration (*β* = 0.114, *t*(21) = 0.462, *p* = 0.649) of diabetes.

**Table 4:** Multiple linear regression analysis of TMV in diabetic participants

TMV	*B*	95% confidence interval		*β*	*t*	*R*^2^	*ΔR*^2^
Diabetic group		Lower limit	Upper limit				
Model						0.281	0.109
Constant	7.456 ***	5.746	9.165		9.070		
HbA_1c_	0.039	− 0.187	0.264	0.072	0.357		
Age	− 0.012	− 0.029	0.005	− 0.569	− 1.475		
Sex (M − F)	− 0.268	− 0.617	0.080	− 0.335	− 1.600		
Type (2–1)	0.359	− 0.284	1.002	0.394	1.161		
Duration	0.005	− 0.018	0.029	0.114	0.462		

The un-standardised regression co-efficient is denoted by *B*; the standardised co-efficient is denoted by *β*; the standardised co-efficient divided by its standard error (SE) is denoted by *t*; the co-efficient of determination is denoted by *R*^2^; the adjusted co-efficient of determination is denoted by Δ*R*^2^

^*^p ≤ 0.05

^**^p ≤ 0.01

^***^p ≤ 0.001

**Table 5:** Multiple linear regression analysis of TMV in non-diabetic participants

TMV	*B*	95% confidence interval		*β*	*t*	*R*^2^	*ΔR*^2^
Non-diabetic group		Lower limit	Upper limit				
Model						0.071	− 0.050
Constant	8.008 ***	5.797	10.219		7.493		
HbA_1c_	− 0.011	− 0.422	0.399	− 0.012	− 0.057		
Age	− 0.004	− 0.012	0.003	− 0.255	− 1.157		
Sex (M − F)	− 0.017	− 0.358	0.325	− 0.022	− 0.102		

The un-standardised regression co-efficient is denoted by *B*; the standardised co-efficient is denoted by *β*; the standardised co-efficient divided by its standard error (SE) is denoted by *t*; the co-efficient of determination is denoted by *R*^2^; the adjusted co-efficient of determination is denoted by Δ*R*^2^

^*^p ≤ 0.05

^**^p ≤ 0.01

^***^p ≤ 0.001

## Discussion

The complication of DCMO affects an estimated 6.8% of the diabetic population [2]. Our study aimed to address this type of maculopathy before it had fully developed. Using SS-OCT, we measured the foveolar thickness at the intersection of the radial scans (CPT). Our group analysis found that diabetic individuals had significantly greater foveolar thickness compared with their age- and sex-matched non-diabetic counterparts (Fig. 2). Increased foveal thickness without accumulation of fluid in cystoid spaces is not yet accorded with any clinical significance: only cystoid oedema is known to cause impaired acuity. This DCMO is treatable with intra-vitreal anti-vascular endothelial growth factor agents [25–27] and corticosteroids [28, 29], both of which reduce inflammation and oedema. However, these are expensive [30], may not entirely restore the quality of sight and carry a risk of the disastrous complication of endophthalmitis [31, 32].

In this explorative study, we have shown a conspicuous positive relationship between CPT and HbA_1c_ in diabetic individuals (*β* = 0.501, *t*(21) = 3.139, *p* = 0.005): for every 1% increase in HbA_1c_ level, there was a 19-μm increase in CPT (Fig. 3). It is known that DCMO is associated with poor glycaemic control [33, 34]. This suggests that the more severe abnormality of DCMO might be preceded by a pre-clinical phase of non-cystoid thickening. Moreover, tight glycaemic control can reduce the propensity for DCMO in diabetic individuals: the UKPDS study found that intensive glycaemic control significantly decreased the development and progression of DCMO [4], and two other large-scale studies found a similar positive effect of tight glycaemic control [3, 5]. In all of these studies, as was best practice at the time, DCMO was identified by means of stereoscopic fundus photography [35]. However, this quasi-quantitative method made it difficult to assess for sub-clinical changes that would have occurred at the macula prior to DCMO becoming clinically evident. This is in contrast to the novel imaging technique of SS-OCT to which we now have access, which permits quantitative assessment of foveal thickness with a resolution in the order of microns.

A previous study found a positive correlation between TMV and glycaemic control [36]: only diabetic participants were included in this study, and one of the inclusion criteria was a duration of diagnosis of diabetes of at least 10 years. Our study aimed to encompass the diabetic population as a whole, with a view to detecting sub-clinical changes at the macula that potentially precurse DCMO; therefore, we included in our diabetic group individuals who had been diagnosed with either type 1 or type 2 diabetes, regardless of the time since diagnosis. In addition, we had an age- and sex-matched non-diabetic control group. Although our correlation between macular thickness and glycaemic control was in agreement with that reported in the previous study [36], we found no correlation between TMV and HbA_1c_ level. TMV encompasses the macular volume across the whole ETDRS grid (Fig. 1), whereas CPT is a measure of foveolar thickness at the intersection of the radial scans. A study using OCT found that the fovea tends to be the area that is most affected [37]: the authors found a statistically significant difference in macular thickness between the diabetic and non-diabetic groups in the central sub-field of the ETDRS grid, but there were no significant differences between groups in the surrounding (superior, inferior, nasal and temporal) areas. The central macula is peculiarly susceptible to oedema because of the foveal avascular zone: this region of human retina, which has the highest cone photoreceptor cell density, is completely devoid of retinal capillaries [38], and the cells within this region receive their blood supply from the choriocapillaris. A more recent study found no significant effects of short- or long-term blood glucose levels on foveal thickness in type 2 diabetes; the authors speculated that vascular permeability was a more effective factor to determine the propensity for increased macular thickness [39].

Analysis of our diabetic sub-groups found that foveolar thickness differed between type 1 and type 2 diabetic sub-groups: on average, individuals with type 1 diabetes had a CPT that was 12 μm greater than that of their type 2 diabetic counterparts, although this difference did not achieve statistical significance (*β* = − 0.223, *t*(21) = − 0.834, *p* = 0.414). There have been numerous epidemiological studies on DCMO in diabetes [40]. In the minority of those studies that have examined the prevalence of DCMO in both type 1 and type 2 diabetic individuals, the evidence has been mixed: some studies have found that this type of maculopathy is more prevalent in type 1 diabetes [2, 41, 42], whereas others have found the opposite [43, 44]. Type 1 diabetes accounts for approximately 5–10% of cases of diabetes, and type 2 diabetes accounts for 90–95% of cases [45]; these disparate prevalence rates can have a considerable effect on statistical power and false positive error rates [46].

Our results of hyperglycaemic diabetic individuals differ from those of an OCT study on non-diabetic individuals who were euglycaemic at baseline, in whom acute hyperglycaemia was induced [47]; the authors found no increase in central macular thickness in any of the participants. Accumulation of fluid at the fovea often develops slowly with few overt symptoms, and although its pathophysiology remains unclear, one of the main risk factors is chronic hyperglycaemia. Diabetic individuals’ glucose levels are generally chronically elevated; therefore, a short period of induced hyperglycaemia in non-diabetic individuals would not necessarily have mimicked a true diabetic response.

We recognise that our study had some limitations. The intra-class correlation co-efficients between measures obtained from participants’ right and left eyes indicated a significant inter-ocular relationship; therefore, we averaged the data from both eyes [23, 24]. One limitation of this statistical approach is that DCMO can present asymmetrically. Most diabetic individuals in this study had relatively good glycaemic control, and the highest HbA_1c_ level recorded from any participant was 9.0%; therefore, future studies should aim to include diabetic participants with elevated or severely elevated HbA_1c_ level. Due to the differences in epidemiology between type 1 and type 2 diabetes [45], it was not possible to achieve equal numbers of individuals with type 1 and type 2 diabetes. Moreover, in order that the results may be generalised to the population and to increase the power of the study, a larger sample size would be required; and this should include individuals of different races. A previous study has found an inverse relationship between participants’ blood pressure and central macular thickness [48]. Given the co-existence of systemic hypertension [49] and dyslipidaemia [50] in the majority of cases of type 2 diabetes (and to a lesser extent in cases of type 1 diabetes), future studies should aim to control for participants’ blood pressure and lipid levels.

The potential for clinical application of our findings will depend upon further longitudinal studies with serial measurement of foveolar thickness, in addition to the current cross-sectional study. Two follow-up studies would be required: one might be a review of retinae that have developed DCMO to see if OCT scans taken prior to its development showed a trend of increasing sub-clinical foveolar thickening that was not noticed at the time, but which can be demonstrated retrospectively; the other should be a longitudinal study of diabetic individuals using frequent SS-OCT to see if a trend of progressive foveolar thickening can culminate in DCMO. If it is demonstrated that non-cystoid foveolar thickening can predict visual impairment, another study would be needed to establish whether tight glycaemic control, for example, by instituting continuous monitoring of blood glucose or use of an insulin pump, can arrest or reverse that trend.

In conclusion, SS-OCT is a useful method to measure foveolar thickness. Indeed, SS-OCT is the only way to quantify this measure in vivo*.* We have found that diabetic individuals have thicker foveolae than non-diabetic individuals. Moreover, in the diabetic group, foveolar thickness appeared to be significantly correlated with participants’ glycaemic control. Our results suggest that, in diabetes, sub-clinical changes may occur at the fovea before DCMO becomes clinically evident. This could provide diabetic individuals with an early warning of disease progression and perhaps motivate them to improve control of their diabetes, with a view to avoiding the need of intra-vitreal injections with their attendant risks.
